# Aberrant lipid metabolism in hepatocellular carcinoma cells as well as immune microenvironment: A review

**DOI:** 10.1111/cpr.12772

**Published:** 2020-01-31

**Authors:** Bo Hu, Jian‐Zhen Lin, Xiao‐Bo Yang, Xin‐Ting Sang

**Affiliations:** ^1^ Department of Liver Surgery Peking Union Medical College Hospital Chinese Academy of Medical Sciences and Peking Union Medical College Beijing China

**Keywords:** fatty acid oxidation, fatty acid synthesis, hepatocellular carcinoma, local immune status, metabolic reprogramming, tumour microenvironment

## Abstract

Hepatocellular carcinoma (HCC) is a primary malignancy of the liver with a high worldwide prevalence and poor prognosis. Researches are urgently needed on its molecular pathogenesis and biological characteristics. Metabolic reprogramming for adaptation to the tumour microenvironment (TME) has been recognized as a hallmark of cancer. Dysregulation of lipid metabolism especially fatty acid (FA) metabolism, which involved in the alternations of the expression and activity of lipid‐metabolizing enzymes, is a hotspot in recent study, and it may be involved in HCC development and progression. Meanwhile, immune cells are also known as key players in the HCC microenvironment and show complicated crosstalk with cancer cells. Emerging evidence has shown that the functions of immune cells in TME are closely related to abnormal lipid metabolism. In this review, we summarize the recent findings of lipid metabolic reprogramming in TME and relate these findings to HCC progression. Our understanding of dysregulated lipid metabolism and associated signalling pathways may suggest a novel strategy to treat HCC by reprogramming cell lipid metabolism or modulating TME.

**Abbreviations**
ACC: acetyl‐CoA carboxylase
AFB1: aflatoxin B1
APCs: antigen‐presenting cells
ACLY: ATP‐citrate lyase
ATGL: adiposite triglyceride lipase
ALOX5: arachidonate 5‐lipoxygenase
APOA4: apolipoprotein A‐IV
ACACB: acetyl‐CoA carboxylase beta
AGPAT9: 1‐acylglycerol‐3‐phosphate O‐acyltransferase 9
Cars: acylcarnitines
COX‐2: cyclooxygenase‐2
CPT2: carnitine palmitoyl transferase 2
CTLA‐4: cytotoxic T‐lymphocyte‐associated protein 4
CSC: enriching cancer stem cells
DCs: Dendritic cells
DRP1: dynamin‐related protein 1
ER: endoplasmic reticulum
FA: fatty acid
FAAs: fatty acid amides
FAO: fatty acid oxidation
FAS: fatty acid synthesis
FASN: fatty acid synthase
FABP1: fatty acid binding protein1
FATP: fatty acid transport protein
FoxP3: forkhead box P3
GARP: repetitions predominant
GS, GLUL: glutamine synthetase
HBx: Hepatitis B virus X protein
HCC: hepatocellular carcinoma
HFSR: hand–foot skin reaction
HDL: High‐density lipoproteins
HDAC3: histone deacetylase 3
HMOX1: heme oxygenase 1
HMg‐CoA: hydroxy‐3‐methyl‐glutaryl‐CoA
HMGCR: hydroxy‐3‐methyl‐glutaryl‐CoA reductase
IRE‐1α: inositol‐requiring protein 1α
JNK: c‐Jun NH2‐terminal kinase
LDs: lipid droplets
LPL: lipoprotein lipase
LPS: lipopolysaccharide
LXR: liver X receptor
MDMs: M2 monocyte‐derived macrophages
MSR1: macrophage scavenger receptor 1
MDSC: myeloid‐derived suppressor cells
MLKL: mixed lineage kinase domain like protein
MUFAs: monounsaturated fatty acids
mTORC2: mammalian target of rapamycin complex2
NASH: nonalcoholic steatohepatitis
NLRP3: pyrin domain‐containing 3
NAFLD: nonalcoholic fatty liver disease
NK cell: natural killer cell
ORFs: overlapping open reading frames
PEp: plasmalogens
PCKS9: proprotein convertase subtilisin kexin 9
PD‐L1: programmed cell death‐ligand 1
PPARs: peroxisome proliferator‐activated receptors
PPARGC1B: PPAR gamma coactivator 1 beta
RCT: randomized controlled trial
RIP3: receptor interaction protein3
ROS: reactive oxygen species
RORγt: retinoic acid‐related orphan receptor gamma t
SCD: stearoyl‐CoA desaturase
STAT6: signal transducer and activator of transcription 6
SLC1A2: solute carrier family 1 member 2
SREBP‐1: sterol regulatory element‐binding protein 1
TAG: triacylglycerol
TAMs: tumor‐associated macrophages
TLR4: toll‐like receptor 4
TME: tumor microenvironment
TNF: tumor necrosis factor
TADCs: tumor associated dendritic cells
Tregs: regulatory T cells
XBP1: X‐box binding protein
7‐DHC: 7‐dehydrocholesterol

## 1 BACKGROUND

**Figure 1:**
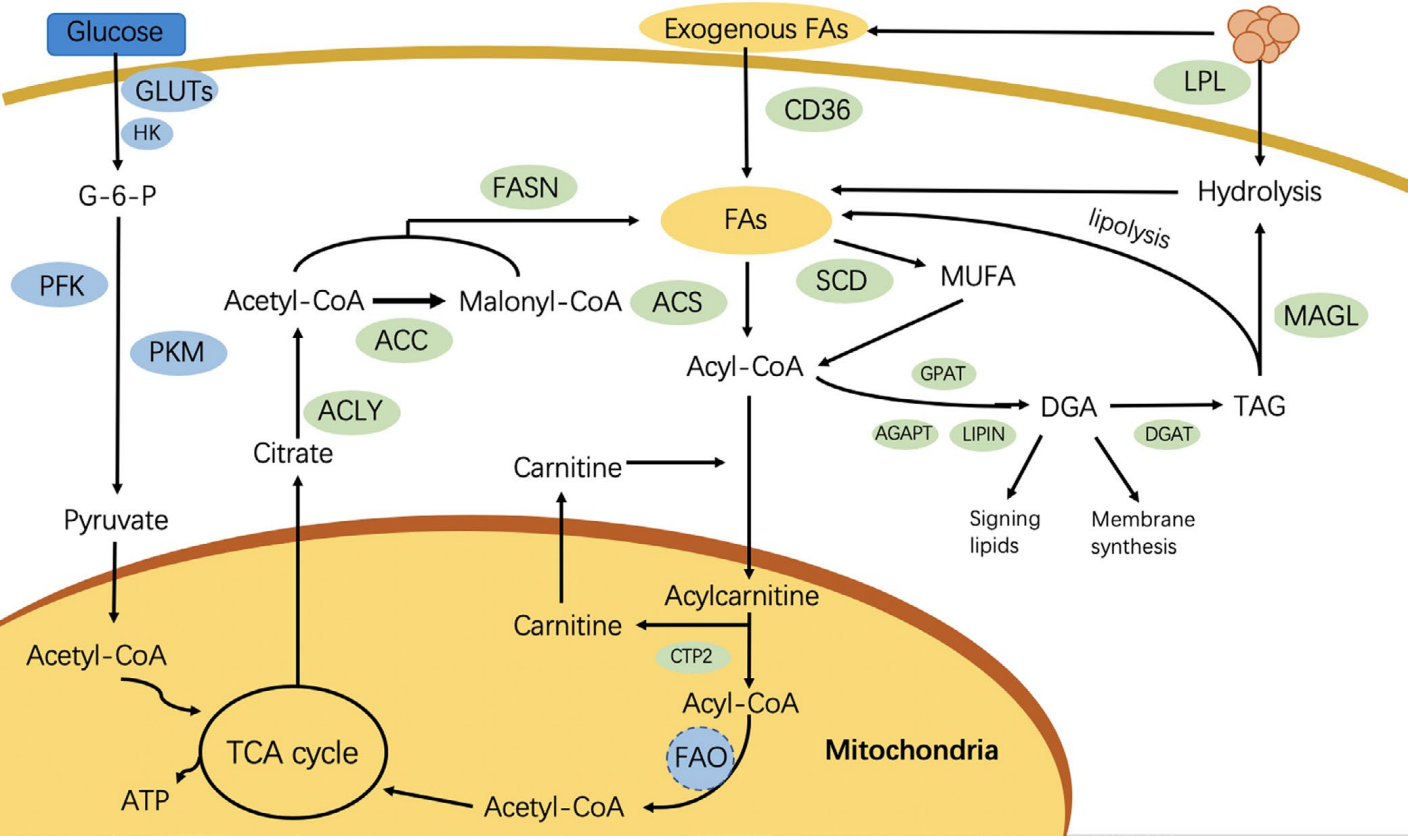
Overview of fatty acid metabolism in hepatocellular carcinoma cells. ACC, acetyl‐CoA carboxylase; ACLY, ATP‐citrate lyase; ACS, acyl‐CoA synthetase; AGPAT, acylglycerolphosphate acyltransferase; CACT, carnitine acylcarnitine translocase; CPT1A, carnitine palmitoyltransferase 1A; CPT2, carnitine palmitoyltransferase 2; DAG, diacylglycerol; DGAT, diacylglycerol acyltransferase; FA, fatty acid; FASN, fatty acid synthase; Gluts, glucose transporters; GPAT, glycerol‐3‐phosphate acyltransferase; MAGL, monoacylglycerol lipase; MUFA, monounsaturated fatty acid; PFK, phosphofructokinase; PKM, pyruvate kinase; SCD, stearoyl‐CoA desaturase; TAG, triacylglycerol; TCA, tricarboxylic acid

Hepatocellular carcinoma (HCC) is the second leading cause of cancer‐related deaths worldwide, and approximately 800 000 cases are diagnosed annually.^1^ There are surgical treatment and chemotherapy for HCC, but the mortality rate remains high. Therefore, it is urgent to further explore the characteristics of HCC and to develop the novel therapies. Tumour microenvironment (TME), which can be hypoxic, acidic and deficient in nutrients, may result in the metabolism of tumour cells and the neighbouring stromal cells, including myeloid cells (such as tumour‐associated macrophages, dendritic cells and myeloid‐derived suppressor cells) and lymphocytes (T cells and B cells) for remodelling, thus facilitating tumour survival, proliferation and metastasis.^2^, ^3^, ^4^ The TME of HCC may involve multiple metabolic abnormalities, among which, abnormal lipid metabolism is a fairly new field that attracts wide attention over the past few years. Dysregulation of lipid metabolism, especially for the metabolism of fatty acid (FA) where the aberrantly activated oncogenic signalling pathways alter the lipid‐metabolizing enzyme expression and activity, has increasingly been recognized as an important metabolic rewiring phenomenon in tumour cells (Figure 1) and immunocytes, and it may also participate in HCC development and progression.^3^ This review aims to examine the mechanism by which this dysregulation modelled HCC cells and neighbouring immunocytes and support HCC progression and to explore the way for therapeutically targeting the aberrant lipid metabolism to benefit the HCC patients.

## 2 ALTERED LIPID METABOLISM OF HEPATOCELLULAR CARCINOMA CELLS

### 2.1 Aberrant lipid metabolism

Increasing evidence suggests that alterations in tumour lipid metabolism, including metabolite abundance and accumulation of lipid metabolic products, lead to tumour development as well as local immunosuppression in the TME.^5^ For instance, a previous study illustrated that the deletion of 5‐lipoxygenase in the TME promoted lung cancer progression and metastasis through regulating T‐cell recruitment.^6^ A recent study has analysed the global gene expression profile of HCC, which reveals that genes involved in the biosynthesis of fatty acids (FAs) are universally up‐regulated in most HCC tissues compared with the noncancerous liver tissues.^7^, ^8^ Typically, FAs function as the signalling molecules, energy sources, and the structural components of cell membrane, all of which are essential for cancer cell proliferation.^9^ Normal cells preferentially utilize the circulating exogenous lipids, whereas cancer cells, including HCC cells, show a high de novo lipid synthesis rate,^10^ suggesting FA accumulation in tumour cells. The roles of major lipogenic enzymes, such as stearoyl‐CoA desaturase (SCD), fatty acid synthase (FASN) and acetyl‐CoA carboxylase (ACC), have been reported to participate in hepatocarcinogenesis. For instance, the genetic ablation of FASN, which is responsible for synthesizing palmitate (C16:0) from acetyl‐CoA and malonyl‐CoA in the presence of NADPH, completely suppresses the Akt‐driven HCC development through inhibiting the Rictor/mammalian target of rapamycin complex2 (TORC2) signalling.^11^ ACC, which converts acetyl‐CoA to malonyl‐CoA as the first rate‐limiting step in de novo lipogenesis, has attracted wide attention as a therapeutic target for non‐alcoholic steatohepatitis (NASH). Typically, ACC1, an isoform of ACC, has been reported by Wang et al^12^ as an independent prognostic indicator for HCC patients, and the ACC1‐driven de novo FA synthesis promotes HCC cell survival, especially under the metabolic stress conditions, like glucose limitation or antiangiogenetic treatment. In addition, recent research has demonstrated that PPARα‐SCD1 axis plays an important role in maintenance of the enriching cancer stem cells (CSC) properties of HCC sphere cells by promoting nuclear accumulation of β‐catenin,^13^ thereby producing novel views for the role of lipogenic enzymes in HCC. Inhibition of SCD1 interferes with sphere formation, down‐regulated expression of CSC‐related markers, and reduces β‐catenin nuclear accumulation.

**Figure 2:**
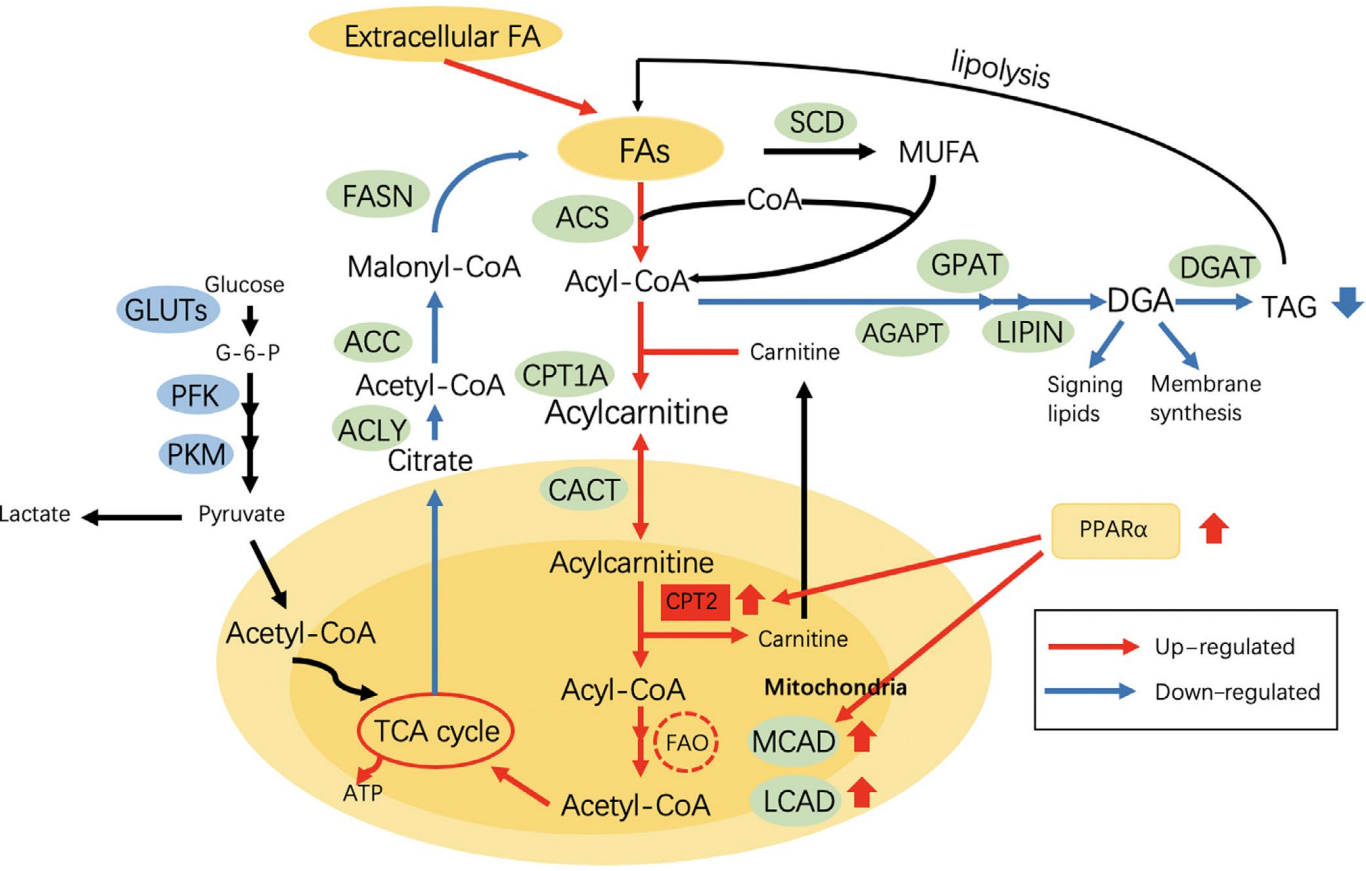
Lipid metabolic reprogramming in β‐catenin‐activated HCC. Fatty acid β‐oxidation (FAO) is activated to fuel HCC

Although reduced FAO has been reported in many cases, some HCCs display a distinctly different metabolic phenotype characterized by a high β‐oxidation rate.^8^ Enhanced FAO, reduced glycolysis accompanied by the up‐regulated expression of PPARα and CPT2 are observed in the β‐catenin‐activated HCCs derived from mice and humans (Figure 2), suggesting that such tumours rely mainly on FAO to provide energy.^8^, ^9^, ^14^ Typically, the β‐catenin‐activated HCCs carry an activating mutation in the *CTNNB1*, a gene that encodes β‐catenin in the Wnt pathway, whose mutation is not uncommon (19.5%) in human HCC.^15^, ^16^, ^17^ Inhibiting FAO by genetic and pharmacological approaches blocks the HCC development, which suggests that inhibiting FAO is a suitable therapeutic approach for the β‐catenin‐mutated HCC.^14^ In addition, Iwamoto et al recently reported that HCC cells would rather utilize FAO for their survival under the hypoxic conditions induced by the antiangiogenic drugs. Their results further showed that the oxygen and nutrient depletion induced by antiangiogenic drugs changed the glucose‐dependent metabolism to the lipid‐dependent metabolism through enhancing free FA uptake and the subsequent FAO, thus stimulating cancer cell proliferation. This phenomenon was mediated by the hypoxia‐induced increased phosphorylation of activated protein kinase (AMPK), which increased FAO via increasing the CPT‐1 activity by inducing the inhibitory phosphorylation of ACC2.^18^

Recently, Bidkhori et al^19^ had identified three HCC subtypes named iHCC1, iHCC2, and iHCC3 and pointed out that tumours in iHCC1 had the highest FAO fluxes, whereas 75% iHCC2 tumours carried mutations in *CTNNB1*, with up‐regulated expression of β‐catenin target genes (like glutamine synthetase GLUL and glutamate transporter SLC1A2). Finally, iHCC3 tumours were associated with the highest fluxes in FA biosynthesis and a strong Warburg effect. Noteworthily, iHCC1 is the tumour group with the highest survival rate, which displays a high inflammation response compared with that of iHCC2 and is potentially associated with type 2 diabetes and obesity. iHCC3 tumours lead to the lowest survival rate and multiple malignant tumour features, including hypoxic behaviour and epithelial‐to‐mesenchymal transition (EMT). Therefore, it is speculated in this study that HCC patients with elevated FAO levels may have better prognosis, which is associated with higher inflammatory and immune responses. In addition, HCC with β‐catenin mutation may be affected by lipotoxicity in a lipid‐rich environment. On the other hand, the expression of phosphorylated AMPK is negatively correlated with the Ki‐67 level (a cell proliferation marker), tumour grade and tumour size in HCC, as mentioned above, thus indicating that the high FAO level mediated by AMPK may be considered as a favourable factor.^20^ In summary, these observations uncover the distinct differences in lipid metabolism within HCC that stem from the high inter‐tumour heterogeneity, which are associated with patient survival.

### 2.2 The Role of sterol regulatory element‐binding protein 1 in regulating lipid metabolism and promoting hepatocarcinogenesis

Furthermore, differential expression of transcription factors (TFs) also serves as the critical element for regulating lipid metabolism. Among them, sterol regulatory element‐binding protein 1 (SREBP‐1), a crucial TF, greatly affects the downstream targeted lipid genes, especially for the downstream target genes in the cholesterol metabolism pathway, which is also a master regulator of SCD, ACC and FASN.[21, 22, 23 Such result indicates that SREBP‐1 up‐regulation promotes the synthesis of FAs related to the up‐regulation of ATP‐citrate lyase (ACLY), FASN and SCD; increases cholesterol uptake into hepatocytes correlated with proprotein convertase subtilisin kexin 9(PCKS9) up‐regulation; and modulates mitochondrial fatty acid oxidation(FAO) associated with acetyl‐CoA carboxylase beta(ACACB) down‐regulation.^5^ Moreover, the large‐scale gene expression profiling conducted by Yamashita et al^24^ revealed marked activation of the SREBP‐1‐mediated lipogenic pathway in HCC and suggested that the up‐regulated SREBP‐1 protein expression was associated with dismal prognosis. Suppressing SREBP‐1 in HCC cells induces growth arrest and apoptosis, whereas over‐expressing SREBP‐1 enhances cell proliferation, suggesting that SREBP‐1 may be a therapeutic target for HCC.^9^

### 2.3 MicroRNAs affect the development of HCC and NAFLD by regulating the lipid metabolic enzymes via the metabolic‐related transcription factors

Recent studies contribute to understanding microRNAs (miRNAs), which are a class of small non‐coding RNAs that regulate gene expression at post‐transcription level and affect the pathogenesis of HCC through regulating the lipid metabolism‐related proteins in liver. For instance, the targeted combination of miRNA1207‐5p with FASN inhibits HCC invasion via inhibiting the Akt/mTOR signalling pathway, whereas FASN up‐regulation reverses the inhibition of miRNA‐1207‐5p on HCC cells.^25^, ^26^ Research conducted by Wu et al^27^ revealed a mechanism by which microRNA‐21, in part, promoted hepatic lipid accumulation and HCC tumour progression by interacting with the HBP1‐p53‐SREBP1c pathway and suggested the potential therapeutic value of microRNA‐21‐anti‐sense oligonucleotide. In addition, miRNA‐3941, miRNA‐4517 and miRNA‐4672 can reduce the degree of hepatic steatosis through inhibiting the expression of fatty acid binding protein 1 (FABP1), thus delaying the progression of non‐alcoholic fatty liver disease (NAFLD), which is a major catalytic agent of HCC.^28^, ^29^ On the other hand, miRNAs also exert crucial roles in regulating the lipid‐metabolizing enzymes through the metabolic‐related transcription factors. A variety of miRNAs, including miRNA‐33a/b, miRNA‐182, miRNA‐96 and miRNA‐24, have been identified to participate in regulating SREBPs.^30^, ^31^, ^32^ MicroRNA‐631 and microRNA‐155 were reported to have a negative regulatory effect on LXRα, which played a key role in regulating FA metabolism by regulating SREBP1‐c as well as the downstream targets involved in FA synthesis.^33^, ^34^ Several miRNAs have been summarized in Table 1.

**Table 1:** Regulation of microRNA to HCC, NAFLD and lipid metabolism‐related transcription factors

microRNA	Way of action	Result	Reference
miR‐1207‐5p	FASN‐mediated Akt/mTOR signalling pathway	Inhibiting HCC	Zhao et al^25^
miR‐30a‐5p	MTDH/PTEN/AKT pathway	Inhibiting HCC	Li et al^26^
miR‐21	HBP1‐p53‐SREBP1c pathway	Promoting HCC	Wu et al^27^
miR‐3941	Inhibiting the expression of FABP1	Inhibiting NAFLD	Wu et al^28^
miR‐4517			
miR‐4672			
miRN631	Repressing LXRα	Inhibiting de novo lipogenesis and LXRα‐induced lipid droplet accumulation	Zhao et al^32^
miR‐155	Repressing LXRα	Inhibiting hepatosteatosis	Miller et al^33^
miR‐24	Down‐regulating the expression of INSIG‐1	SREBP activation	Wu et al^32^
miR‐182	Down‐regulating the expression of FBXW7	Negatively affecting nuclear SREBP accumulation	Jeon et al^30^
miR‐96	Down‐regulating the expression of INSIG‐2	Negatively affecting nuclear SREBP accumulation	Jeon et al^30^

Abbreviations: FABP1, fatty acid binding protein 1; FASN, fatty acid synthase; FBXW7, F‐box and WD repeat domain‐containing 7; HBP1, HMG‐box protein 1; HCC, hepatocellular carcinoma; INSIG, insulin‐induced gene 1; LXRα, liver‐X‐receptor α; MTDH, metadherin; NAFLD, non‐alcoholic fatty liver disease; PTEN, phosphatase and tensin homolog; SREBP, element‐binding protein; SREBP1c, sterol regulatory element‐binding protein 1c.

### 2.4 Uptake and transport of exogenous FAs in HCC

Although most cancer cells exhibit a metabolic shift towards lipogenesis and synthesize nearly all esterified FAs de novo, some tumours tend to “acquire” free FAs directly from the external environment.^35^ External FAs are brought into the cell through a transport mechanism involving specialized enzymes and proteins such as FA translocase (FAT or CD36), fatty acid transport proteins FATP2 and FATP5, and members of FABP family (FABP1, FABP4 and FABP5), which are involved in FA uptake and transport in hepatic tissues as well as in HCC.^36^, ^37^ Increased expression of CD36 leads to higher FA uptake in HCC, which is closely related to induction of the mesenchymal transition (EMT).^36^ The EMT may also be promoted when the enhanced FA levels of HCC patients up‐regulate inflammation‐related oncogenic transcriptional factors (NF‐κB, AP‐1, STAT3 and HIF‐1α), which activate Wnt and TGF‐β signalling pathways.^36^, ^38^, ^39^ Given that depletion of FASN has been reported to significantly suppress HCC development,^40^ there is also a great demand for novel therapeutic targets involved in lipid uptake and transport. In mouse hepatocytes, adenovirus‐mediated knockdown of FATP2 or genetic deletion of FATP5 has been reported to significantly decrease the rates of fatty acid uptake,^41^, ^42^ which might have the potential to be new targets with regard to HCC. Taken together, more researches are needed to study more detailed mechanisms and verify the effect of deletion.

### 2.5 Liver dysfunctions linked to HCC

**Figure 3:**
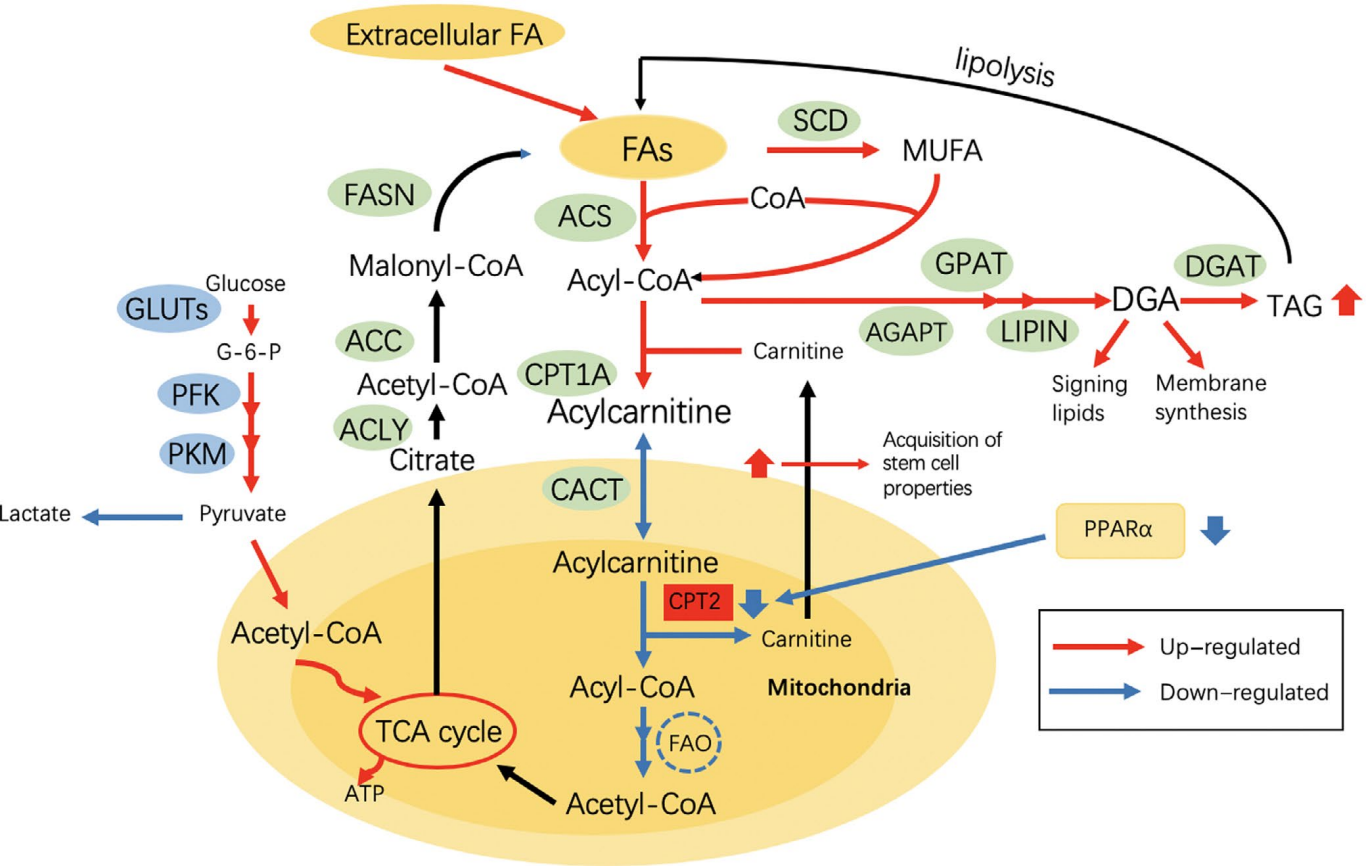
Lipid metabolic reprogramming in obesity‐ and non‐alcoholic steatohepatitis (NASH)‐related HCC. Fatty acid β‐oxidation (FAO) is suppressed for adaptation to a lipid‐rich environment

Liver cancer displays a high incidence among people with chronic non‐infectious liver diseases. Specifically, a non‐esterified FAs‐rich condition may be a characteristic environment of obesity‐ and non‐alcoholic steatohepatitis (NASH)‐driven HCC (Figure 3).^43^, ^44^ Nonetheless, it remains to be further explored about how HCC cells survive and grow in such an environment. Research shows that in human steatohepatitic HCC (SH‐HCC), the expression of carnitine palmitoyltransferase 2 (CPT2), which converts acylcarnitine back to acyl‐CoA, is down‐regulated; subsequently, the marked accumulation of acylcarnitine species is detected, suggesting that the serum acylcarnitine levels may serve as a biomarker of HCC.^45^ More importantly, CPT2 down‐regulation suppresses the FAO pathway and enables HCC cells to escape from lipotoxicity to adapt to a lipid‐rich environment, which is achieved through inhibiting the Src‐mediated c‐Jun NH2‐terminal kinase (JNK) activation.^46^ Furthermore, oleoyl carnitine (AC18:1), the long‐chain acylcarnitine that accumulates through FAO suppression induced by CPT2 down‐regulation, enhances hepatocarcinogenesis through the signal transducer and activator of transcription 3 (STAT3)‐mediated acquisition of stem cell properties.^9^, ^46^ On the other hand, peroxisome proliferator‐activated receptors (PPARs) are reported to be involved in regulating mitochondrial metabolism in liver within the disease context from NASH to HCC. The altered PPARs expression mainly induces mitochondrial metabolic dysfunctions, which can suppress FA oxidation, accumulate reactive oxygen species (ROS), and promote of lipogenesis.^47^ The above evidence demonstrates that concomitant liver diseases may affect lipid metabolism and further promote tumour progression.

## 3 INFLUENCE OF HEPATITIS VIRUSES ON HCC DEVELOPMENT

Hepatitis viruses, especially for hepatitis B virus (HBV) in Eastern countries and hepatitis C virus (HCV) in Western countries, are another major factor in HCC occurrence and development, which can lead to corresponding metabolic alterations.^48^ Typically, the HBV X protein (HBx), which is one of the four overlapping open reading frames (ORFs) in the HBV genome, is involved in the development of HBV‐associated HCC. Teng et al^49^ observed that the lipid (including triglycerides, cholesterol and FAs) profiles revealed a biphasic response pattern during the progression of HBx tumorigenesis; in this response pattern, a small peak at an early phase correlated with the oxidative stress (OS) and pro‐inflammatory response, together with a large peak or terminal, switched at the tumour phase. It is worth mentioning that the abnormal hepatic FAs levels result in the synergistic induction of HBx protein and liver inflammatory gene expression, which is achieved through HBx protein stabilization.^50^ Furthermore, five lipid metabolism‐related genes have been identified, including the arachidonate 5‐lipoxygenase (*ALOX*5), lipoprotein lipase (*LPL*), *FABP*4, 1‐acylglycerol‐3‐phosphate O‐acyltransferase 9 (*AGPAT*9) and apolipoprotein A‐IV (*APOA*4), which are remarkably activated in the HBx transgenic HCCs and are further validated in human HBV‐related HCCs. Inhibiting these lipid genes reverses the effect of HBx on lipid biosynthesis and suppresses the HBx‐induced cell proliferation in vitro.

It is also reported in literature that the combination of HBx with aflatoxin B1(AFB1) exposure increases cyclooxygenase‐2 (COX‐2) expression to mediate the up‐regulation of receptor interaction protein 3 (RIP3) and dynamin‐related protein 1 (DRP1), which in turn promotes the localization of necrosomes of the RIP3‐mixed lineage kinase domain like protein (MLKL) on mitochondria, subsequently exacerbating steatosis in hepatocytes.^51^, ^52^ Another potential mechanism is elucidated as that liver FABP1 expression is dramatically increased in the sera of HBV‐infected patients, as well as in both sera and liver tissues of HBV‐transgenic mice, which promotes hepatic lipid accumulation. Xu et al also suggested that HBx induced lipid accumulation in liver tissues through decreasing the amount of lipases penetrating into lipid droplet, or inhibiting the enzymatic activity through activating CDC42 and PPARγ, which further aggravated the inflammatory reaction and eventually led to HCC. On the other hand, the HBx‐depressed miR‐205 and miR‐384, are elaborated to be responsible for the abnormal lipid metabolism through accumulating cholesterol and mediating de novo lipid synthesis in HCC cells.^53^, ^54^, ^55^ In another model, the HBx‐induced lipogenesis and HCC development may also involve the Ras family oncogene Rab18, and its expression can be up‐regulated by pathways related to COX‐2 and miR‐429, thus increasing the lipid anabolism.^56^ However, HBx is also reported to promote HCC survival through inducing FAO and increasing the intracellular ATP and NADOH levels, and this can induce the resistance to glucose deprivation through activating the AMPK and FAO pathways in HCC cells.^57^, ^58^

In terms of HCV‐related HCC, the HCV core protein has been reported to play a vital part in enhancing the transcriptional activities of SREBP1 and PPARγ, and stimulating the expression of lipogenic enzymes and FAs uptake associated proteins. In other words, FA synthesis and uptake are up‐regulated in HCV, and such up‐regulation is further enhanced in HCC. Meanwhile, genes involved in FAO are down‐regulated in both HCV and HCC groups.^59^, ^60^, ^61^ In another study, the expression levels of haem oxygenase 1 (HMOX1) and CPT1, which is indicative of the mitochondrial beta‐oxidation, are shown to be up‐regulated in FA‐stimulated cells, and such increase is remarkably higher in HCV^+^ than in HCV^−^ cells.^62^ Additionally, Lange et al^63^ investigated the associations between CYP2R1, GC, and DHCR7 genotypes that are determinants of reduced 25‐hydroxyvitamin D (25[OH]D3) serum levels and the risk of HCV‐related HCC development and then pointed out that genetic variations in the three loci were associated with progression to HCC in patients with chronic hepatitis C. Their data suggested a functionally relevant role for vitamin D in the prevention of HCV‐related hepatocarcinogenesis. Due to the prevalence of vitamin D deficiency, scholars have also studied the methods of improving vitamin status and found a substantial dose‐dependent increase of non‐hydroxylated vitamin D in the liver of mice fed a diet containing 7‐dehydrocholesterol (7‐DHC), which provided the evidence that dietary 7‐DHC seemed to affect vitamin D metabolism.^64^ Interestingly, the intracellular levels of 7‐DHC or its derivatives can have deleterious effects on cellular functionality and viability, and Gelzo et al^65^ have demonstrated that 7‐DHC could exert its cytotoxic effects on cancer cells, which associated with an increase in Bax levels, decrease in Bcl‐2/Bax ratio, reduction of mitochondrial membrane potential, increase in apoptosis‐inducing factor levels, unchanged caspase‐3 activity, and absence of cleavage of PARP‐1. However, the relationship between 7‐DHC, vitamin D and HCV‐related HCC needs to be further explored. To sum up, the effect of hepatic viruses on the lipid metabolism in HCC is a complex process, and more studies on the mechanism of hepatitis virus‐induced abnormal lipid metabolism should be carried out, in order to explore the role of aberrant lipid metabolism caused by hepatitis virus in the initiation and development of hepatitis virus‐related HCC.

## 4 LIPID METABOLIC REPROGRAMMING OF IMMUNOCYTES IN HCC

**Figure 4:**
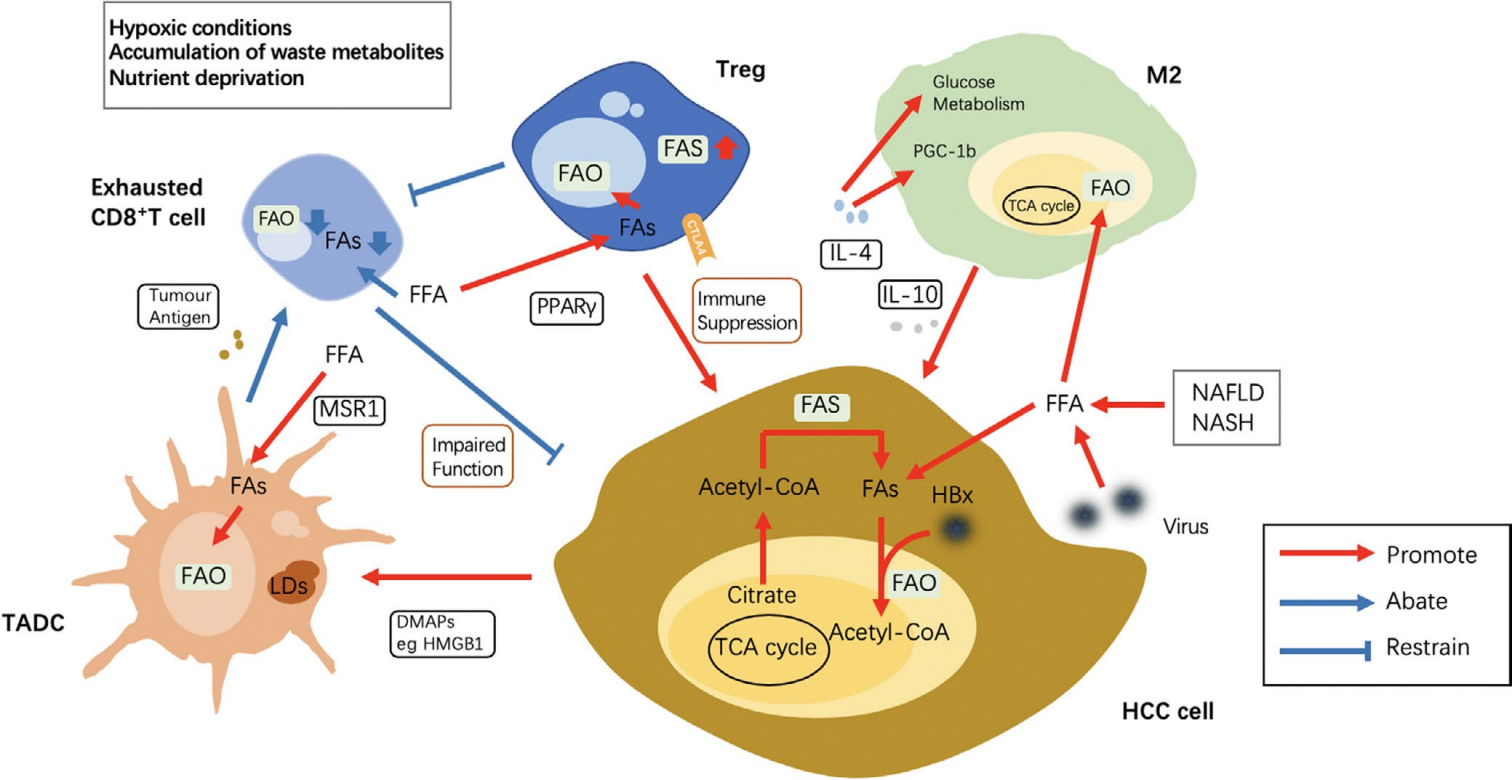
Lipid reprogramming in the tumour microenvironment affects the anti‐/pro‐tumoral functions of immune cells. Different immune cells in the TME of HCC exhibit different lipid metabolism changes, which affect their functions. These metabolically reprogrammed immune cells then have a differed influence on the liver cancer cells compared to the original immune cells. FAO, Fatty acid oxidation; HCC, Hepatocellular carcinoma; TME, tumour microenvironment

The role of lipid metabolism in regulating immune cells has recently aroused general concerns. Evidence collected in several types of solid tumours indicated the importance of tumour immunometabolic reprogramming and suggested a novel and crucial area for future research of liver cancer.^48^ The complicated crosstalk between metabolically reprogrammed immune cells and liver cancer cells has been suggested, but the molecular mechanisms need further exploration (Figure 4).

### 4.1 Tumour‐associated macrophages

Macrophages are the versatile innate immunocytes, which contribute to diverse situations, including host defence, homeostasis and pathology. They can be induced to the M1 and M2 phenotypes according to the surrounding microenvironment. The former is pro‐inflammatory and polarized by either lipopolysaccharide (LPS) alone or in combination with Th1 cytokines, like IFN‐γ and GM‐CSF, and it is characterized by the production of inflammatory cytokines, tumour necrosis factor (TNF), reactive nitrogen and oxygen intermediates, and microbicidal functions. By contrast, the latter is anti‐inflammatory and immunoregulatory, which can be polarized by Th2 cytokines like interleukin 4 (IL‐4) and IL‐13, and produce the release of anti‐inflammatory cytokines, such as IL‐10 and TGF‐β.^66^, ^67^

In a tumour setting, macrophages undergo changes in their lipid profile. Among them, tumour‐associated macrophages (TAMs) are the predominant M2 phenotype in solid cancers including HCC.^68^ Zhang et al had utilized an in vitro model to mimic the TAM‐HCC interaction in TME, and their results suggested that the M2 monocyte‐derived macrophages (MDMs) promoted HCC cell migration in an FAO‐dependent manner by enhancing IL‐1β secretion. Besides, they further put forward that IL‐1β induction was ROS and pyrin domain‐containing 3 (NLRP3)‐dependent.^69^, ^70^ Previous study also demonstrated that FA was consumed in the M2‐like TAMs resulted from the IL4‐driven activation of signal transducer and activator of transcription 6 (STAT6) and PPARG coactivator 1 beta (PPARGC1B), which peaked with the increases in mitochondrial biogenesis and epigenetic reprogramming towards FAO.^71^ Besides, triacylglycerol (TAG) uptake was also found to be essential for FAO in M2 macrophages, which was orchestrated by PPAR and liver‐X‐receptor (LXR).^72^, ^73^

In addition, apparently at odds with the increased FAO utilization, some TAMs accumulate intracellular lipids, which support not only their metabolic fitness, but also their immunomodulatory functions. Moreover, it has also been demonstrated that lipid loading of macrophages is associated with increased tumoricidal and inflammatory capacities.^71^, ^74^ Likewise, fatty acid synthesis (FAS) enzymes are up‐regulated in M2‐polarized macrophages, and the de novo synthesized FAs are at least partially used to feed back into FAO.^73^, ^75^ As for the murine peritoneal macrophages, an increased intracellular lipid content was shown to be linked with an enhanced cytotoxic activity, especially in those that were artificially enriched in polyunsaturated FA relative to those enriched in cholesterol.^74^ In another study, the FAS expression in TAMs is suggested to polarize these cells to an IL‐10‐expressing pro‐tumour phenotype.^76^ In addition, the E‐FABP‐expressing TAMs are also reported to produce the high levels of IFN‐β, which is essential for recruiting natural killer (NK) cells and anti‐tumour activity through up‐regulating the formation of lipid droplets (LDs).^74^, ^77^ Nevertheless, accumulation of TAMs in HCC is linked with dismal prognosis.^78^ Importantly, these macrophages express the immune checkpoint protein programmed cell death‐ligand 1 (PD‐L1), together with other immunosuppressive signals, like Toll‐like receptor 4 (TLR4) and CD48/2B4, which inactivate the CD8^+^ T cells, promote the recruitment of regulatory T cells (Tregs) and suppress the activity of NK cells, thereby affecting local immunity.^79^, ^80^, ^81^, ^82^ More studies should be performed on the effect of abnormal lipid metabolism of TAM on its immune function and tumour progression in HCC.

### 4.2 T cells in TME

Recent studies have established that metabolic restrains, such as glucose restriction, impair the activities of effector T cells in TME.^69^, ^83^ In the same context, the remarkable expansion of activated Treg cells, which is characterized by the expression of CD4, CD25, cytotoxic T lymphocyte‐associated protein 4 (CTLA‐4) and forkhead box P3 (FoxP3) in tumour tissues, has been described in both mice and humans, thereby contributing to the suppression of protective anti‐tumour immunity.^48^, ^84^ CD8^+^ T cells are the most important executors of the anti‐tumour adaptive immunity, including HCC. Compared with normal liver, tumour tissue has a lower CD8^+^ T cells density whereas a higher Tregs density, which indicates dismal prognosis.^48^ According to previous studies, the exhausted CD8^+^ T cells infiltrating in HCC are marked by the down‐regulated expression of multiple FASs and the decreased intracellular FAs content, as well as functional exhaustion linked with reduced interferon‐γ secretion while up‐regulated PD‐1 expression.^85^, ^86^ The intracellular FAs content plays a crucial part in the levels of FAO and oxidative phosphorylation in cells, among which the former is a keyway for immunocytes to produce ATP.^87^, ^88^ Lipids are known to be the essential materials for cells, and their depletion in CD8^+^ T cells dramatically inhibits cell proliferation and signal transduction, which partly explains the lower number of CD8^+^ T cells in HCC than in adjacent tissues. In terms of the related mechanism, the above‐mentioned abnormal FAs metabolism in CD8^+^ T cells may be related to the inactivation of the SREBP pathway.^89^ Moreover, SREBP deficiency hinders the metabolic reprogramming towards a glycolytic phenotype, which is typical for T‐cell activation. Alternatively, Treg cells, which activate AMP‐activated protein kinase and mainly rely on FAO rather than glycolysis, can survive under the amino acid and nutrient depletion conditions induced by the high glycolytic activity of proliferating tumour cells coupled with the poor vasculature within HCC, thereby exerting their immunosuppressive effects.^7^, ^90^, ^91^ As indicated in open literature sources, the advantage of Tregs over conventional T cells (Tconvs) in TME may be dependent on the capacity of Tregs cells to compete for glucose and perform FAS and FAO at the higher rates than those of Tconvs. Additionally, it is proposed that FAS, rather than FA uptake, shapes the lipid Treg pool and contributes to Treg proliferation, after considering the high neutral lipid content and the metabolite signature observed in tumour bed Tregs.^83^, ^92^, ^93^

Furthermore, Kalathil et al^94^ pointed out that the frequency of expression of intracellular glycoprotein A repetitions predominant (GARP) and CTLA‐4 in CD4^+^ Foxp3^+^ Tregs was also markedly increased among HCC patients compared with that in controls. However, CTLA‐4 inhibits glycolysis without augmenting FAO, suggesting that CTLA‐4 sustains the metabolic profile of non‐activated cells, which potentially indicates that CTLA‐4 makes no contribution to the increase in FAO in tumour‐associated Tregs.^95^ Meanwhile, Treg cells also express PD‐1, which promotes the FAO of endogenous lipids through up‐regulating CPT1A expression and inducing lipolysis, as indicated by the elevated levels of lipase adipocyte triglyceride lipase (ATGL), the lipolysis marker glycerol and the release of FAs.^95^, ^96^ However, PD1 expression level in HCC‐related Tregs should be further investigated. Moreover, it is also suggested in research that the frequency of PD‐1^+^ CD4^+^ T cells and PD‐1 expression levels in HCC patients are evidently higher than in healthy donors, which potentially implies that the above‐mentioned PD1 regulation of FAO may be reflected in such cells.^94^

On the other hand, natural killer T (NKT) cells, which share characteristics with both adaptive and innate immune cells and have multiple immunoregulatory roles, are CD1d restricted T cells that mostly recognize lipid antigens.^97^ Since NKT cells mostly recognize lipid antigens, an altered tumour lipid metabolic profile will also alter the repertoire of lipid antigens that can potentially affect their immune‐modulatory function.^98^ One study in an obese mouse model for NAFLD reported a reduction in the number of hepatic NKT cells, as a result of activation‐induced death of NKT cells by activated Kupffer cells due to lipid excess.^99^ It was further documented that lipid excess in high‐fat diet (HFD)‐induced obese mice activates type I NKT cells and skews the balance towards a pro‐inflammatory cytokine environment. Moreover, lipid excess also causes hepatic steatosis in an NKT‐dependent manner and can be reversed by deficiency of either type I NKT cells or CD1d.^100^ However, in context of HCC, another study demonstrated that there were no significant changes in the NKT cell number in the background of increased lipid content in the liver with regard to a mouse model,^101^ which inspired us to conduct more in‐depth experiments to compare the effects of lipid changes on NKT cells in the two diseases that have been linked.

In terms of T helper (Th) cells, Endo et al performed transcriptional profiling of memory phenotype CD4 T cells in high‐fat‐fed mice and identified ACC1 as an essential regulator of Th17 cell differentiation in vitro and of the pathogenicity of Th17 cells in vivo and then pointed out that ACC1 could modulate the DNA binding of retinoic acid‐related orphan receptor gamma t (RORγt) to target genes in differentiating Th17 cells.^102^ However, in terms of HCC and other liver diseases, there are few studies on the lipid metabolic reprogramming of Th cells. In the future, more studies should shed light on the effect of abnormal lipid metabolism on Th cell function and the role of Th cell with abnormal lipid metabolism in the development of HCC.

### 4.3 Tumour‐associated dendritic cells

Dendritic cells (DCs) are the professional antigen‐presenting cells (APCs), which bridge innate immunity with the adaptive one. DCs from HCC patients have an impaired ability to trigger immune response, in the meantime of promoting immunosuppression.^103^ Modulating the lipid metabolism, such as de novo FAS during DCs activation, affects endoplasmic reticulum (ER) and Golgi expansion, thereby impacting their antigen‐presenting ability.^104^ In a setting of HCC, the tumour‐associated dendritic cells (TADCs) express the scavenging receptor, such as macrophage scavenger receptor 1(MSR1) that facilitates lipid uptake and accumulation, which seems to support the immunogenic immune responses and cross‐presentation. However, it in turn leads to the low expression of co‐stimulatory molecules and DCs‐related cytokines, as well as the decreased ability to effectively stimulate the allogeneic T cells of TADCs.^105^, ^106^ Interestingly, Herber et al^105^ believed that the above‐mentioned effect was observed in type 1 conventional DCs (cDC1s) and type 2 conventional DCs (cDC2s), but not in plasmacytoid DCs (pDCs), which might reflect the different functions and/or metabolic pathway usages among DC subsets in vivo. Remarkably, the intratumoral infiltration by pDCs is reported as a novel indicator of the dismal prognosis for HCC patients, which is possibly achieved through inducing an immune tolerogenic and inflammatory TME comprising regulatory T and IL‐17‐producing cells.^98^

The differential effect of lipid accumulation in DCs observed in HCC and other tumour settings may be ascribed to the accumulation and/or signalling by the modified lipid species.^107^ According to Wei et al, the oxidized lipids contained in TADCs also affected the cross‐presentation, and they subsequently gave an example to illustrate that the accumulation of oxidized polyunsaturated FAs, cholesterol esters and TAG impaired cross‐presentation without altering the presentation of endogenous antigens. Meanwhile, the accumulation of non‐oxidized lipids made no difference to the cross‐presentation, which suggested that, not only the storage of lipids, but also the accumulation of modified lipids, altered the DC function.^108^

Recently, the role of TADCs in LDs formation has attracted considerable attention. The bioenergetic and synthetic demands for DCs to fluctuate extensively, and the build‐up of intracellular LDs, may reflect a recent surplus of energy, and an actively changed metabolic programme.^109^ Cubillos‐Ruiz et al^110^ deemed that tumour‐derived factors triggered lipid peroxidation in TADCs, which activated the endoplasmic reticulum (ER) stress response mediated by the inositol‐requiring protein 1α(IRE‐1α) as well as its target X‐box binding protein 1 (XBP1). At the same time, XBP1 activation can in turn induce a lipid biosynthetic programme, which results in the accumulation of LDs and blunted antigen presentation, leading to a reduced ability to control tumour growth. Taken together, the TME, including HCC, helps to shape the functional phenotype of DCs in the presence of LDs, and predicts a great diversity of LD types in healthy and ill subjects. However, the detailed mechanism and role of LDs formation in DC cells should be further investigated.

### 4.4 Other immune cells

Apart from the above‐mentioned immunocytes, abnormal lipid metabolism is also reflected in the myeloid‐derived suppressor cells (MDSC), neutrophils and natural killer (NK) cells, which may contribute to tumour progression. The increased FAs uptake and FAO have been demonstrated to regulate the immunosuppressive function of tumour infiltrating MDSCs, which is accompanied by the elevated mitochondrial mass, up‐regulated key FAO enzymes and increased oxygen consumption rate.^111^, ^112^ Additionally, FAS has also been reported to have some relevance in neutrophil biology. In this regard, Lodhi et al^113^ showed that the synthesis of peroxisomal lipid drove inflammation by supporting the phospholipid composition and viability of neutrophil membrane. However, there are few reports about the changes in lipid metabolism within these cells in the context of HCC. Therefore, future studies are warranted to shed light on the types and mechanisms of lipid abnormalities in the HCC immune microenvironment.

## 5 PERSPECTIVES OF HCC THERAPY FROM LIPID METABOLIC REPROGRAMMING

From another point of view, serum lipids exert their functions to become a novel biomarker of HCC. Lu et al^114^ investigated the diagnostic and prognostic potential of serum lipid signatures for HCC, and further illustrated that plasmalogens (PEp), which were based on triglycerides and phosphatidylethanolamine, displayed a fair capability to discriminate HCC patients from the healthy controls; besides, they were remarkably associated with the HCC grades and were thereby identified as the potential biomarkers of HCC. In another study regarding the efficacy of sorafenib chemotherapy in HCC, Saito et al^115^ put forward their views that the lower levels of lipids containing FA (18:2) were related to the positive responses, whereas the lower levels of acylcarnitines (Cars) and fatty acid amides (FAAs) resulted in the susceptibility to sorafenib‐induced hand‐foot skin reaction (HFSR), which had revealed the novel candidate biomarkers. In summary, abnormal lipid can potentially serve as a biomarker for diagnosing HCC.

As mentioned above, some HCC cells and immune cells recruited from TME meet their demands for energy and building materials by up‐regulating the FAS, which have led the researchers to target key lipogenic enzymes, particularly FASN, or related metabolites, to slow down tumour growth.^116^ For instance, TVB‐2640, the latest orally available FASN inhibitor, is currently used in clinical trials to treat HCC.^117^ Nonetheless, several first‐generation FASN inhibitors exhibit strong toxicity in preclinical and clinical trials; therefore, targeting histone deacetylase 3 (HDAC3) may provide a new strategy to target FASN.^118^ Noteworthily, *Fasn* ablation markedly delays but not prevents hepatocarcinogenesis in mice, whereas the concomitant inhibition of FASN‐mediated FAS and hydroxy‐3‐methyl‐glutaryl‐CoA (HMg‐CoA) reductase (HMGCR)‐driven cholesterol production is highly detrimental for HCC cell growth in culture.^119^ In addition, some studies report that the increased SCD activity promotes hepatocarcinogenesis through accumulating monounsaturated fatty acids (MUFAs) and activating the unfolded protein response via the ER stress related to sorafenib resistance in HCC. In addition, the researchers believe that SCD inhibition may reshift the balance of FA composition towards saturation, which exert a synergistic effect on HCC with sorafenib.^120^ Besides, inhibition of FAO by etomoxir or other methods (such as targeted delivery of CPT1A siRNA/shRNA) may limit the immunosuppressive function of M2 macrophages, which may be developed as an effective therapeutic strategy.^48^ Some immunotherapies related to lipid metabolism for cancers were summarized in Table 2. Nonetheless, in consideration of the genotypic and tumour‐biological diversities of HCC patients, as well as the complex lipid metabolism, therapeutic strategies targeting the FA‐related pathways against HCC still lag behind.

**Table 2:** Immunotherapy related to lipid metabolism for cancers

Targets	Agents	Mechanisms	Developments
CPT1A	Etomoxir	Inhibition of FAO	Preclinical
CTLA‐4	Ipilimumab	Checkpoint blockade; inhibition of FAO	FDA‐Approved
PD‐1/PD‐L1	Nivolumab, Pembrolizumab, Atezolizumab 2	Checkpoint blockade; inhibition of FAO	FDA‐Approved
AMPK	Metformin	Increased FAO	FDA‐Approved

Abbreviations: AMPK, adenosine monophosphate‐activated kinase; CPT1A, carnitine palmitoyltransferase 1‐a; CTLA‐4, cytotoxic T lymphocyte antigen 4; FAO, fatty acid β‐oxidation; PD‐1, programmed cell death‐1.

## 6 CONCLUSIONS

Our understandings towards the lipid metabolic changes in HCC development have been improved remarkably over the past years. Nevertheless, the impacts of dysregulated FAs metabolism on HCC cells and the TME remains incompletely known So far here, the characteristics of lipid metabolism in HCC cells and surrounding immune cells are summarized, with the focus on the FA synthesis, oxidation and uptake pathways. Typically, the complicated lipid metabolic network, which regulates various functions of immunocytes, can either be functionally oriented or environmentally adapted; nonetheless, the two can together lead to immune microenvironment homeostasis, which affects HCC progression. These observations suggest that lipid metabolic reprogramming constitutes an essential part for the tumorigenicity of these cells, which may be critical for cancer stem cells, particularly during the HCC initiation and invasion processes. In future research, clinical development of miRNA therapeutics should be concerned. For instance, an miRNA specifically targeting miR‐33a/b, which plays a regulatory role in cellular cholesterol transportation, can increase circulation level of high‐density lipoproteins (HDL)‐cholesterol by enhancing randomized controlled trial (RCT) to the plasma, liver and faeces, and reduced plaque size and lipid content in mice.^121^, ^122^, ^123^ More attention should be paid to miRNA therapeutics for HCC with aberrant lipid metabolism. Afterwards, as we mentioned above, studies on exogenous lipid uptake and transport should also be further carried out to obtain new therapeutic targets in an HCC setting. In addition, the mTOR pathway may be a suitable target to regulate immune cells by manipulating cellular lipid metabolism. Inhibition of mTOR by rapamycin can block the development of macrophages and CD4^+^ T cells in several scenarios, including liver cancer.^124^, ^125^ Future studies can make efforts to combine immunity and lipid metabolism, so as to develop novel therapeutic methods that can benefit patients with HCC.

## CONFLICT OF INTEREST

The authors declare that they have no competing interests.

## AUTHORS' CONTRIBUTIONS

XS and JL created the idea for the review. BH performed the selection of literature, drafted the manuscript, and prepared the figures. XY and JL revised the manuscript. All authors read and approved the final manuscript.

## CONSENT FOR PUBLICATION

All authors agree to submit for consideration for publication in the journal.
